# Genotype Diversity of Highly Pathogenic Avian Influenza H5N1 Clade 2.3.4.4b in Pennsylvania Poultry During Disease Outbreak from April 2022 to March 2023

**DOI:** 10.3390/v18050502

**Published:** 2026-04-28

**Authors:** Deepanker Tewari, Manoj K. Sekhwal, Chrislyn Nicholson, Mary L. Killian, Corey Zellers, Julia Livengood, Kristina Lantz, Mia Torchetti, Alex Hamberg

**Affiliations:** 1 Pennsylvania Veterinary Laboratory, Harrisburg, PA 17110, USA; 2 Department of Veterinary and Biomedical Sciences, Penn State University, State College, PA 18432, USA; 3 Animal and Plant Health Inspection Service, Harrisburg, PA 17110, USA; 4 National Veterinary Services Laboratories, Ames, IA 50010, USA; 5 Pennsylvania Department of Agriculture, Harrisburg, PA 17110, USA

**Keywords:** HPAI, Avian Influenza, Genotype, Pennsylvania, Poultry, Clade 2.3.4.4b, Outbreak, H5N1

## Abstract

The 2022 highly pathogenic avian influenza (HPAI) outbreak of H5N1 clade 2.3.4.4b was one of the major avian influenza outbreaks, leading to multiple spillover events infecting domestic and wild bird flocks, as well as mammals. The sustained spread was a result of viral circulation in wild birds across migratory flyways in North America. Pennsylvania has a significant poultry population that supports both retail and live bird markets. The state also features migratory bird stopovers on the Atlantic flyway, increasing exposure to HPAI infections. This study investigates clinical presentation and sequence data from H5N1 clade 2.3.4.4b viruses during the 2022 outbreak in Pennsylvania. Eight different H5N1 clade 2.3.4.4b genotypes were detected (A1, B1.1, B1.2, B1.3, B2.2, B3.3, B3.5, and one minor genotype) during the first year. The earliest detection was genotype A1, a fully Eurasian virus, in commercial poultry in April 2022. All other genotypes identified were reassortants of A1 with North American avian influenza gene segments (denoted with “B”). Genotype B3.3 was a rare genotype prior to the initial spillover into the live bird market system, but remained predominant among backyard flocks in Pennsylvania and surrounding states until September 2023. Genotype B3.3 has not been detected in migratory waterfowl since, suggesting the genotype has waned and is no longer in circulation. This study sheds light on the genotype diversity of H5N1 during the 2022 outbreak in Pennsylvania poultry, contributing to the understanding of virus evolution and its potential impacts.

## 1. Introduction

The highly pathogenic avian influenza virus (HPAI) of H5N1 clade 2.3.4.4b was first detected in the United States (U.S.) from wild waterfowl in South Carolina in December of 2021 after circulating in European countries since October 2021 [1]. Since the first detection of H5N1 clade 2.3.4.4b, it has disseminated to over 150 wild bird species across nearly every U.S. state, resulting in prolific mass mortality events [2]. The sustained virus circulation has contributed to viral spread across all four migratory flyways in North America, allowing for genetic reassortment with existing low pathogenic avian influenza viruses (LPAIs) to occur in wild birds [1,2,3]. These extensive reassortment events, potentially coupled with the low fidelity of viral RNA-dependent RNA polymerase during viral replication, have led to the emergence of over 100 genetic constellations to date (https://github.com/USDA-VS/GenoFLU version 1.04 accessed on 1 December 2024). This outbreak, declared as one of the largest avian influenza outbreaks in history, has involved multiple spillover events that have impacted both mammals and avian species [1,2,3,4,5,6].

Outbreaks of HPAI have significant impacts for commercial and backyard poultry. The virus causes high morbidity and mortality rates in poultry; in addition, to control the spread of disease, bird flocks are culled, and poultry sales are restricted or suspended. The first detection of the 2022 HPAI outbreak in domestic poultry in the U.S. occurred in February 2022 in Indiana [3,4]. Since then, over 175 million birds have been lost due to mortality or culling. causing significant economic losses [2]. Due to improved biosecurity measures after the 2015 HPAI outbreak, there is limited predicted lateral spread of the 2022 HPAI virus in domestic poultry. The overall epidemiology combined with phylogenetic analysis of the virus suggests that the majority of virus detections in poultry and non-poultry flocks in the U.S. continue to be consistent with independent introductions from wild birds [1,2,3].

Poultry is an important agricultural sector in Pennsylvania, generating over $2 billion in revenue in 2022 (https://www.ers.usda.gov/topics/animal-products/poultry-eggs/sector-at-a-glance/ accessed on 1 December 2024). Pennsylvania is also part of the Atlantic Flyway, and is a pivotal stopping point for many migratory birds. Disease-spread models of the 2022 HPAI outbreak predicted that the southeast Pennsylvania area was at greater risk of future infections due in part to wild bird migrations and to the increased number of backyard flocks that arose during the 2020 COVID pandemic [2,3]. Due to these factors, it is important to evaluate the impact of H5N1 on both commercial and backyard poultry populations. In this study, we describe the clinical signs and the genotypes of H5N1 clade 2.3.4.4b detected in Pennsylvania’s commercial and backyard flocks from April 2022 to March 2023, with additional focus on one specific genotype that was mainly detected in backyard and poultry flocks that supplied live bird markets.

## 2. Materials and Methods

### 2.1. Sample Collection and Viral Whole-Genome Sequencing

Pooled swab samples (up to 11 tracheal swabs for gallinaceous birds, and five cloacal swabs for anseriforms (ducks) were collected from commercial and backyard poultry premises for influenza A virus (IAV) screening as part of the national surveillance program, as well as zone surveillance implemented as per the USDA HPAI Response Plan Red Book. Samples were submitted to the Pennsylvania Animal Diagnostic Laboratory System (PADLS) and tested for IAV using a real-time reverse-transcription PCR (rRT-PCR) that targets the matrix (M) gene, as well as subtype assays including H5 2.3.4.4b. Non-negative samples by these assays were forwarded to the USDA, National Veterinary Services Laboratories (NVSL; National Centers for Animal Health, Ames, IA) for confirmatory testing and characterization. Next-generation sequencing of the viruses across all eight IAV segments (PB2, PB1, PA, HA, NP, NA, M, and NS) and analyses were conducted by NVSL [1,7]. For flocks that showed viral detection, clinical signs were documented for infected flocks, and post mortem was not conducted for birds showing viral detection.

### 2.2. H5N1 Genotyping, Sequence Data Collection, and Genomic Analysis

A total of 175 IAV (H5N1) whole genome sequences were obtained from Pennsylvania outbreaks in domestic poultry during 16 April 2022 to 14 March 2023. For this study, sequences were genotyped using GenoFLU version 1.04 as previously described [1]. Among these sequences, 61 were identified as genotype B3.3 (Supplementary Table S1).

Time to most recent common ancestor analysis (TMRCA) was conducted for B3.3 genotype sequences, including those detected even later than the study period for Pennsylvania. For TMRCA analysis, clade 2.3.4.4b, genotype B3.3, avian hosts, and all geographic locations were retrieved from the GSAID database covering 2022–2024. A total of 93 influenza A virus nucleic acid sequences retrieved from GSAID were included, representing the PB2, PB1, PA, HA, NP, NA, M, and NS gene segments, including a sequence from a red-tailed hawk (23-009488-001) that was detected during the study period in Pennsylvania; this sequence lacked the PB2 segment. All eight gene segments were concatenated for the TMRCA analysis.

In addition, a similar analysis was performed independently for each gene segment (PB2, PB1, PA, HA, NP, NA, M, and NS) using avian influenza A virus nucleotide sequences retrieved from GISAID. For each individual segment analysis, a total of 93 sequences were included. However, for the PB2 segment, 92 sequences were analyzed because the red-tailed hawk strain (23-009488-001) did not contain the PB2 segment in the database. Each sequence was represented by gene segments linked to its corresponding EPI accession IDs. To assess nucleotide similarity, a BLAST version 2.160+ -based identity analysis was performed by comparing all retrieved sequences to a Pennsylvania reference isolate (23-008369-007). Multiple sequence alignment (MSA) was performed using MAFFT version 7.526 [8], and the aligned dataset was processed through the Nextstrain Augur version 32.1.0 (https://docs.nextstrain.org/projects/augur/en/stable/ accessed on 1 December 2024) pipeline to reconstruct a time-scaled phylogeny. First, a maximum likelihood tree was generated with the Augur tree. The tree was refined with augur refine, integrating metadata to estimate branch lengths and infer temporal structure, producing a dated phylogeny and node-specific branch-length data. The refined tree and metadata were exported using augur export v2 to create a JSON file for visualization in Auspice version 2.67.0 (https://auspice.us/ accessed on 1 December 2024). The final time-scaled tree was visualized with ggtree [9] in R version 4.5.2 (https://www.r-project.org/).

vSNP analysis was also performed as outlined previously, using 107 B3.3 sequences; for comparisons, 35 detections from outside Pennsylvania were also included, to better understand viral evolution and spread. The vSNP pipeline produces easy-to-read SNP matrices, sorted for convenience, as well as corresponding phylogenetic trees, making the output easily understandable [10].

## 3. Results

### 3.1. H5N1 Infection and Clinical Signs in Poultry

**Figure 1:**
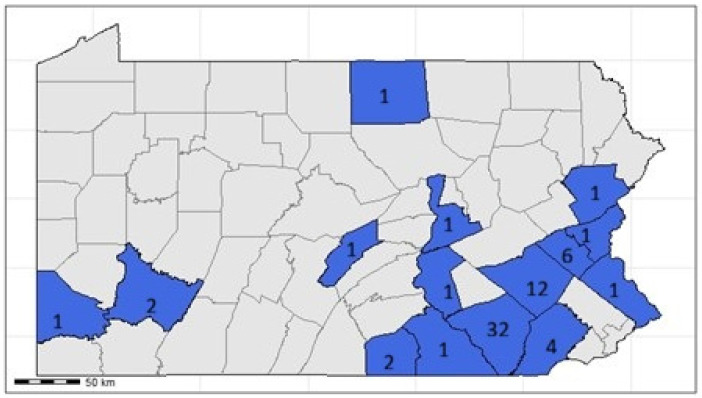
Counties in Pennsylvania where HPAI H5N1 clade 2.3.4.4b was detected in poultry between April 2022 and March 2023. Virus was detected in fifteen counties (shown in color with # of detections labeled for each county), including 32 sites in Lancaster, 12 sites in Berks, 12 sites in Lehigh, 4 sites in Chester, 2 sites each in Allegheny and Adams, and one site each in Northampton, Washington, Westmoreland, Monroe, Dauphin, Tioga, Northumberland, Bucks and Mifflin.

During this period, HPAI H5N1 clade 2.3.4.4b was detected in both commercial and backyard poultry flocks across 15 counties in Pennsylvania (Figure 1). A total of 67 commercial and backyard flocks, including both single-species operations (chickens, ducks, and turkeys) and multi-species operations, were impacted. These premises included 10 commercial layer and broiler operations, 11 turkey farms, 10 duck premises, all with multiple houses and 36 mixed-species backyard premises. The majority of the infected flocks were detected in the Lancaster (32), Berks (12), Leigh (6) and Chester (4) counties. A total of 42,695 PCR tests were conducted, screening approximately 451,303 birds, pooling swabs as per USDA collection guidance, to identify cases and monitor the spread of the virus during the outbreak.

Clinical signs of H5N1 infection in chickens ranged from increased mortality, depression, decreased egg production, and nasal discharge, to no clinical signs observed (at least three premises with no clinical signs). Ducks exhibited clinical signs such as increased mortality, neurological signs, diarrhea, decreased water consumption, decreased egg production, respiratory signs, depression, and, in some cases, no clinical signs (five premises with no clinical signs). Turkeys showed increased mortality or showed no clinical signs (one site with no clinical sign). Backyard flocks (mainly ducks, but also mixed species including ducks, chickens, turkeys, geese, guineas, and quail) displayed either no clinical signs (three flocks with no signs) or signs which included respiratory signs like conjunctivitis, wheezing, and nasal discharge, as well as increased mortality, lethargy, diarrhea, neurological signs, decreased egg production, and reduced feed consumption. The clinical signs observed were strongly influenced by the timing of avian influenza virus (IAV) detection within the flock. Euthanasia was typically performed within 24 h of diagnosis, often prior to the onset of clinical signs or when signs were minimal. Following the establishment of a Highly Pathogenic Avian Influenza (HPAI) Control Zone and the implementation of mandatory weekly AIV surveillance, cases were identified rapidly, with fewer clinical signs observed. Producers were required to promptly report poultry exhibiting clinical signs of IAV to the Pennsylvania Department of Agriculture and/or USDA Veterinary Services.

### 3.2. H5N1 Genotyping and Genotype B3.3 Genome Analysis

**Figure 2:**
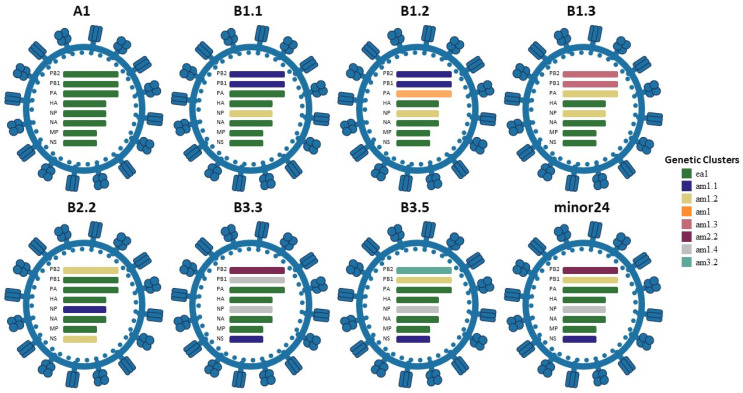
Schematic of the reassortment of the HPAI H5N1 viral gene segments detected in Pennsylvania. Segment lineages North American (am) or Eurasian (ea) are also represented.

**Figure 3:**
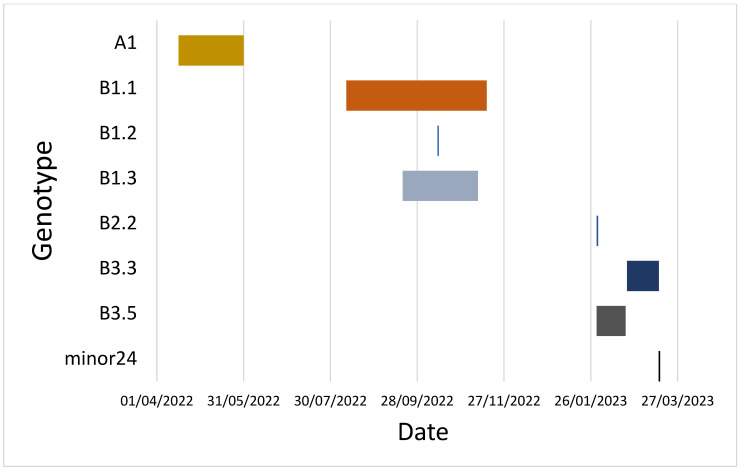
Circulation of highly pathogenic avian influenza genotypes for H5N1 clade 2.3.4.4b in Pennsylvania between April 2022 and March 2023 in domestic poultry. Presence of different viral genotypes is depicted with color and date range.

Using GenoFLU for IAV reassortment and genotyping assignment, 175 sequences from bird samples were analyzed; these were collected from 60 of the 67 premises in Pennsylvania where H5N1 2.3.4.4b was initially detected by rRT-PCR from PADLS laboratories. Eight different genotypes were identified: A1, B1.1, B1.2, B1.3, B2.2, B3.3, B3.5, and minor24 (Figure 2 and Figure 3) (Table 1 and Supplementary Table S1 also provide references to all the 175 sequences that are available publicly). Among the sequences included for analysis, genotypes A1 and B3.3 were overrepresented, with 57 (representing 21 unique premises) and 61 sequences (representing 14 backyard and 3 commercial flocks), respectively. Among all the sequences analyzed, chickens, ducks, turkeys, geese, and guineas were the affected poultry types, with viral detections from ducks and chickens being the most prevalent. Genotype B3.3 is a four-gene North American reassortant of the A1 genotype with American (am) PB2, PB1, NP, and NS segments (Figure 2). Genotype B3.3 spanned only a month but, compared to other genotypes other than the A1 genotype, showed the highest level of infection (Figure 3). With regard to the dominant genotypes, we found 17 infected flocks with the B3.3 genotype: 11 in Lancaster county, four in Chester county, and two in Bucks, along with 21 infected premises: 11 in Lancaster county and 10 in Berks county (Supplementary Table S1). For genotyping analysis, Pennsylvania viral sequences where whole genome sequence was not available, including viral detections at seven premises, were excluded from the GENOFLU analysis.

**Figure 4:**
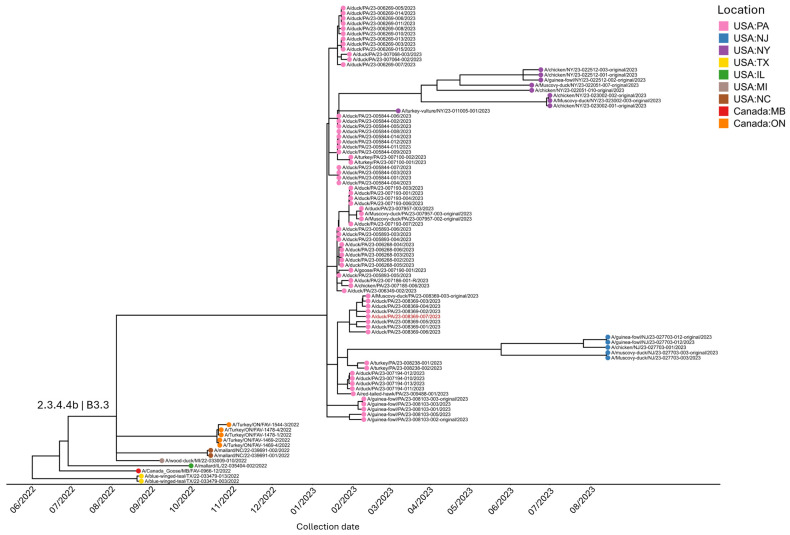
Phylogenetic analysis of HPAI H5N1 isolates. The tree shows the evolutionary relationships of 93 influenza A virus isolates after curation of eight genomic segments (HA, NA, M, NP, NS, PA, PB1 and PB2), PB2 segment was excluded in the case of the red-tailed hawk (23-009488-001). Segment alignments generated using MAFFT version 7.526 [8] and time-scaled phylogenetic reconstruction performed with the Nextstrain Augur pipeline reveal that all isolates cluster within clade 2.3.4.4b, genotype B3.3. Pennsylvania sequences are shown with gray nodes; other locations are shown with colored nodes. A/duck/PA23-008369-007/2023 (in red color) was used as reference strain.

For the TMRCA analysis of genotype B3.3, phylogenetic analysis of eight concatenated segments of genomes (HA, NA, M, NP, NS, PA, and PB1) revealed a uniform evolutionary pattern among HPAI H5N1 viruses circulating in North America, with all isolates clustering together within the genotype. Across the dataset, all isolates showed >99% nucleotide identity and 100% query coverage, including the red-tailed hawk sequence (23-009488-001) which also exhibited > 99% identity to the reference strain (23-008369-007; genotype B3.3) and was collected 3 March 2023 during the poultry B3.3 event in Pennsylvania (Figure 4). The TMRCA analysis demonstrates that early B3.3 genotypes from 2022 cluster together and represent at least three flyways with viruses from Canada, Illinois, Michigan, North Carolina, and Texas. The 2023 Pennsylvania B3.3 virus, which rapidly spread among ducks, geese, chickens, turkeys, and guineas in a short time span, clusters separately from these earlier viruses. The 2023 genotype B3.3 cluster includes viruses from New York, New Jersey and Virginia, indicating strong regional connectivity, which is further supported by the vSNP analysis.

Phylogenetic analyses of each of the eight gene segments conducted separately (HA, NA, M, NP, NS, PA, PB1, and PB2) further confirmed these findings (Supplementary Figures S1–S8). All viral genomes showed over 99% identity for all genes where data was available, including with the virus sequence from a Pennsylvania red-tailed hawk (23_009488_001_2023); this is not unexpected, as the hawk was collected 3 March 2023 during the poultry B3.3 event in Pennsylvania.

**Figure 5:**
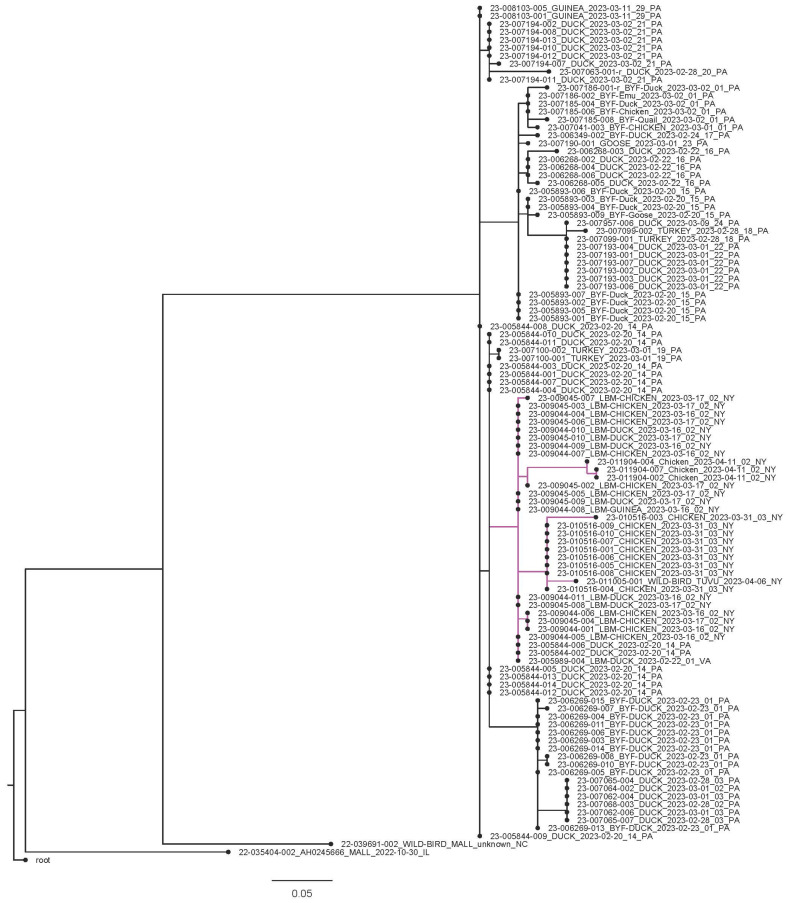
vSNP analysis and phylogenetic tree produced by Galaxy phylogenetic reconstruction with the RaXML tool, showing sequence similarity among B3.3 viruses recovered.

vSNP analysis and phylogenetic analysis (Figure 5 and Supplementary Table S2) along with TMRCA analysis (Figure 4) further substantiated that there was rapid expansion of B3.3 in Pennsylvania among the poultry population including in the backyard duck, chicken, emu, quail, and commercial guinea, duck, chicken and turkey flocks. The data review and analysis also showed multiple flocks being infected within a short span and with the same genotype and presence of distinct clusters within the genotype. (Figure 5). The virus was only detected in one scavenging (vulture) and one raptor species and the genotype B3.3 has not been detected in wild migratory birds since the poultry event ended. vSNP analysis of B3.3 sequences identified clusters of virus representing live bird markets in New York and Virginia as well as Pennsylvania (Figure 5, Supplementary Table S2).

## 4. Discussion

The 2022 outbreak of highly pathogenic avian influenza (HPAI), caused by the H5N1 clade 2.3.4.4b strain, stands as one of the largest in history. The outbreak has caused substantial economic losses due to the culling of infected poultry, and has even impacted some mammals [2,3,5,6]. Additionally, the outbreak has raised concerns about health risks to agricultural workers, adding to its widespread impact. Pennsylvania’s large poultry industry and numerous backyard flocks increase the state’s vulnerability, with major bird migration routes further complicating disease control efforts. Monitoring and mitigating HPAI are crucial for safeguarding agricultural economies and global biosecurity. This study examined H5N1 clade 2.3.4.4b sequence data and clinical information from Pennsylvania’s commercial and domestic flocks, focusing on IAV genotype circulation between April 2022 and March 2023, particularly the predominant genotype B3.3 during 2023.

The clinical signs observed in Pennsylvania’s flocks infected with H5N1 clade 2.3.4.4b were consistent with those outlined by the USDA for HPAI infections (https://www.aphis.usda.gov/livestock-poultry-disease/avian/avian-influenza accessed on 1 December 2024). Chickens exhibited increased mortality, depression, reduced egg production, nasal discharge, or no clinical signs. Ducks displayed increased mortality, neurological signs, diarrhea, respiratory issues, with few or no clinical signs shown in mild cases. Turkeys primarily showed increased mortality or had no clinical signs. Mixed backyard flocks demonstrated respiratory signs, lethargy, neurological signs, reduced egg production, and decreased feed consumption. Monitoring mortality rates, water consumption, and egg production serves as a vital indicator for promptly detecting disease issues within the flock [2]. The lack of widespread clinical signs in most cases was likely due to early detection through effective surveillance and epidemiological investigations. These findings emphasize the crucial importance of surveillance monitoring and proactive strategies for effectively managing H5N1 outbreaks in commercial and backyard flocks.

The genotyping analysis of H5N1 sequences from Pennsylvania indicated the presence of multiple genotypes, including A1, B1.1, B1.2, B1.3, B2.2, B3.3, B3.5, and minor24 (Table 1). Genotypes A1 and B3.3 were detected most frequently among poultry. The infections observed in poultry peaked during the spring months of 2022 (A1) and 2023 (B3.3) in Pennsylvania, in alignment with the timing of migratory bird movements where the risk of point source introductions is highest. The genotype B3.3 event was consistent with initial wild bird exposure followed by lateral transmission among poultry, further spreading the virus in Pennsylvania and later its detection in poultry in the neighboring states. Once the B3.3 event in poultry had ended there was no evidence of onward circulation in wild migratory bird species; there were only two detections in scavenging (vulture) and raptor species during the circulation of this genotype in poultry.

The detection and onward transmission of genotype B3.3 in Pennsylvania poultry population in early spring of 2023 impacted at least 14 backyard flocks and three commercial flocks. The B3.3 sequences demonstrated high similarity (≥99% pairwise identity) across multiple viral segments indicating limited genetic variation between the strains from various locations [11,12,13]. This emphasizes the important role of biosecurity practices in farming and production to avoid infectious disease spillover events.

## 5. Conclusions

In conclusion, the 2022–2023 HPAI outbreak in Pennsylvania significantly affected poultry populations and agriculture. The correlation between infection peaks and migratory bird movements in the spring indicates that migratory birds, particularly those within the Atlantic flyway, played a central role in the initiation of viral spread in Pennsylvania. The detection of genotype B3.3 outside of Pennsylvania highlights the need for enhanced biosecurity and continued focus on effective testing and monitoring strategies, especially for birds destined for live bird markets. Overall, this study provides valuable information on the genotype diversity of H5N1 during the 2022 Pennsylvania outbreak, emphasizing the need for continued surveillance and monitoring to mitigate future risks to poultry and livestock and public health.

## Figures and Tables

**Table 1:** Genotypes and sample collection date ranges of highly pathogenic avian influenza (HPAI) of H5N1 clade 2.3.4.4b detected in Pennsylvania between April 2022 and March 2023 in domestic poultry, based on genetic sequences and genotyping using GenoFLU.

Genotype	Genotype Clusters								# of Seqs	# of Premises	Collection Date Range
	PB2	PB1	PA	HA	NP	NA	M-	NS			
A1	ea1	ea1	ea1	ea1	ea1	ea1	ea1	ea1	57	21 C ^1^	16 April 2022–31 May 2022
B1.1	am1.1	am1.1	ea1	ea1	am1.2	ea1	ea1	ea1	4	2 B ^2^	10 August 2022–15 November 2022
B1.2	am1.1	am1.1	am1	ea1	am1.2	ea1	ea1	ea1	6	1 B	29 January 2023
B1.3	am1.3	am1.3	am1.2	ea1	am1.2	ea1	ea1	ea1	29	7 C, 6 B	18 September 2022–9 November 2022
B2.2	am1.2	ea1	ea1	ea1	am1.1	ea1	ea1	am1.2	4	1 B	30 January 2023
B3.3	am2.2	am1.4	ea1	ea1	am1.4.1	ea1	ea1	am1.1	61	3 C, 14 B	20 February 2023–14 March 2023
B3.5	am3.2	am1.2	ea1	ea1	am1.4.1	ea1	ea1	am1.1	10	1 C, 3 B	30 January 2023–19 February 2023
minor24	am2.2	am1.2	ea1	ea1	am1.4.1	ea1	ea1	am1.1	4	1 B	14 March 2023

^1^ C—Commercial production premises. ^2^ B—Backyard premises. # of sequences (Seqs) collected from # of premises for genotype analysis (Supplementary Table S1).

## Data Availability

The authors confirm that the genetic data supporting the findings of this study for Pennsylvania avian influenza genetic sequences are available in publicly maintained repository, either through GISAID or as NCBI Bio project SRA, as outlined in Supplementary Table S1.

## Supplementary Materials

The following supporting information can be downloaded at: https://www.mdpi.com/article/10.3390/v18050502/s1, Table S1: GenoFLU results; Table S2: Vsnp analysis of avian influenza isolates genotype B3.3: The vSNP analysis and accompanying SNP tables display closely related isolates and identification of mixed SNPs; Figure S1: Hemagglutinin (HA) phylogenetic tree. Time-scaled maximum-likelihood phylogenetic tree of the HA gene segment from HPAI H5N1 viruses retrieved from the GISAID database within clade 2.3.4.4b (genotype B3.3). All isolations are from 2022 to 2024. Pennsylvania isolates are shown with their relative positions to isolates from other U.S. states and Canada; Figure S2: Neuraminidase (NA) phylogenetic tree. Time-scaled maximum-likelihood phylogenetic tree of the NA gene segment; Figure S3: Matrix Protein (M) phylogenetic tree. Time-scaled maximum-likelihood phylogenetic tree of the M gene segment; Figure S4: Nucleoprotein (NP) phylogenetic tree. Time-scaled maximum-likelihood phylogenetic tree of the NP gene segment; Figure S5: NS (Non-Structural) phylogenetic tree. Time-scaled maximum-likelihood phylogenetic tree of the NS gene segment; Figure S6: PA (Polymerase Acidic) Phylogenetic tree. Time-scaled maximum-likelihood phylogenetic tree of the PA gene segment; Figure S7: PB1 (Polymerase Basic 1) Phylogenetic tree. Time-scaled maximum-likelihood phylogenetic tree of the PB1 gene segment; Figure S8. PB2 (Polymerase Basic 2) Phylogenetic tree. Time-scaled maximum-likelihood phylogenetic tree of the PB2 gene segment.
