# Supplementary material for: Genotype Diversity of Highly Pathogenic Avian Influenza H5N1 Clade 2.3.4.4b in Pennsylvania Poultry During Disease Outbreak from April 2022 to March 2023
Source: Viruses. 2026 Apr 28;18(5):502. doi: 10.3390/v18050502 (PMC13211549; doi:10.3390/v18050502)
Supplement: Supplementary file 1 [file viruses-18-00502-s001.zip › Viruses26v1supplMDPI.pdf]

viruses

Supplementary file

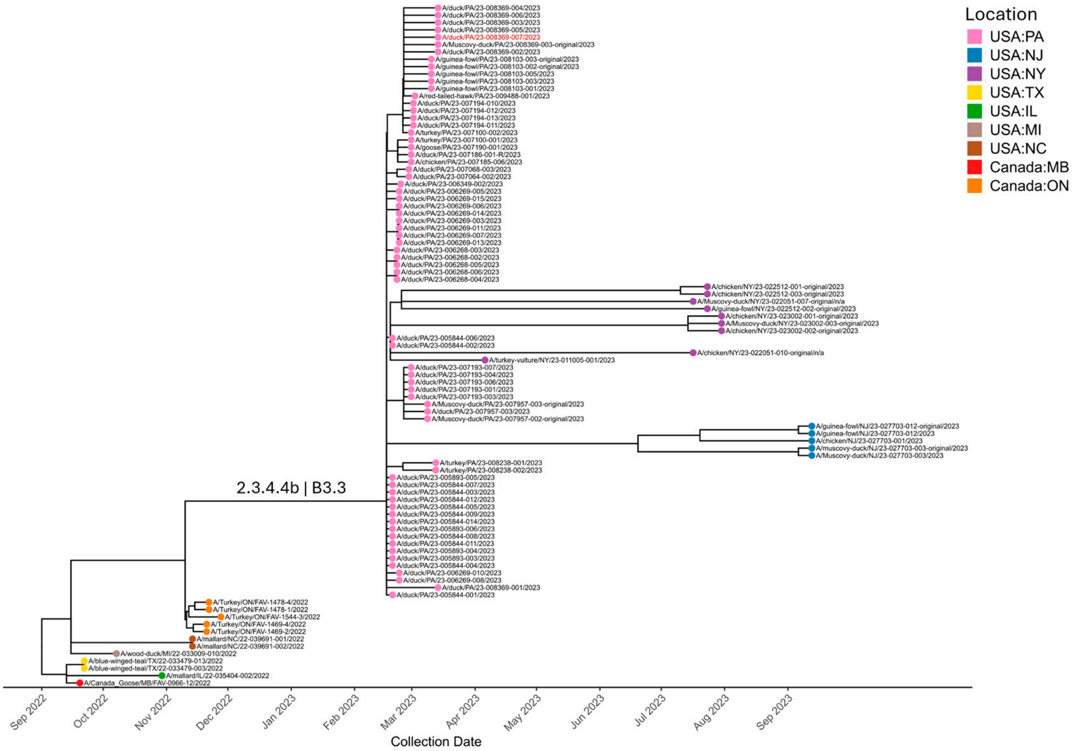

Supplementary **Figure S1. Hemagglutinin (HA) phylogenetic tree.** Time-scaled maximum-likelihood phylogenetic tree of the HA gene segment from HPAI H5N1 viruses retrieved from the GISAID database within clade 2.3.4.4b (genotype B3.3). All isolations are from 2022 to 2024. Pennsylvania isolates are shown with their relative positions to isolates from other U.S. states and Canada. The node label in red shows the reference isolate (23-008369-007).

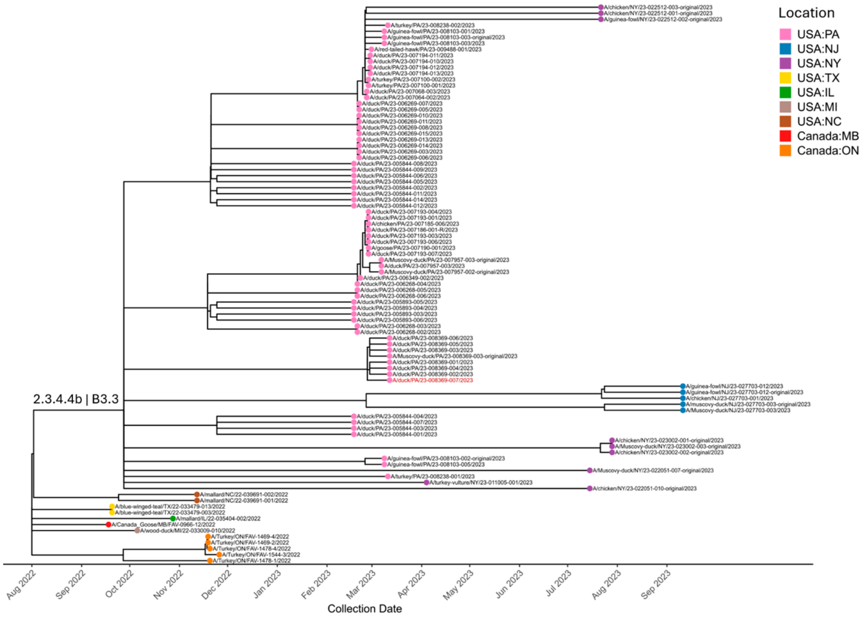

Supplementary Figure S2. **Neuraminidase (NA) phylogenetic tree.** Time-scaled maximum-likelihood phylogenetic tree of the NA gene segment. The node in red shows the reference isolate (23-008369-007).

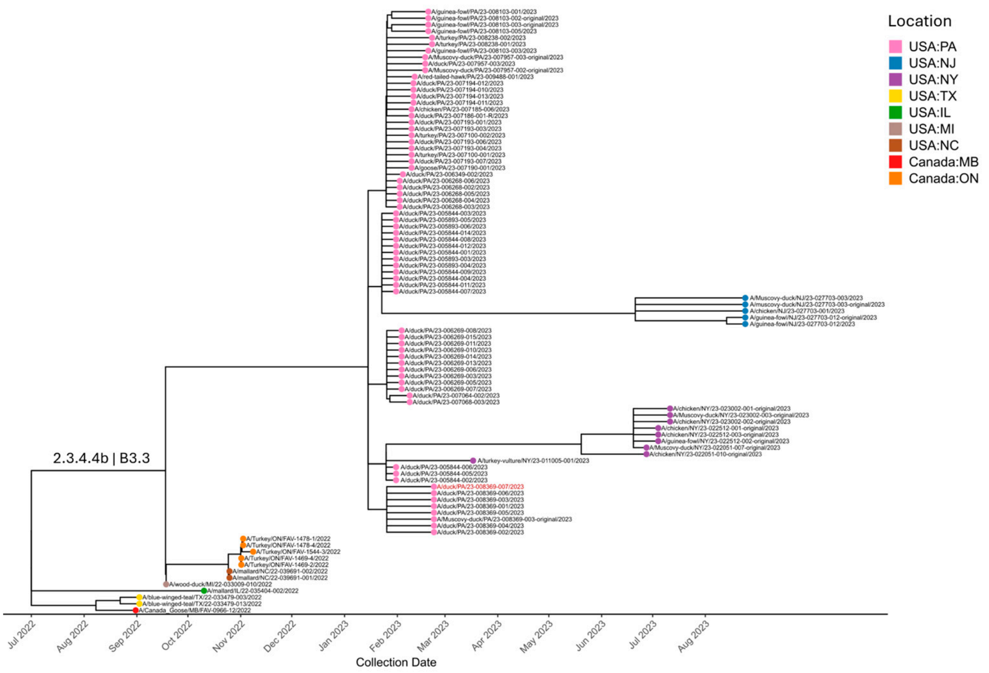

Supplementary Figure S3. **Matrix (M) phylogenetic tree.** Time-scaled maximum-likelihood phylogenetic tree of the MP gene segment.The red color label indicates the reference isolate (23-008369-007).

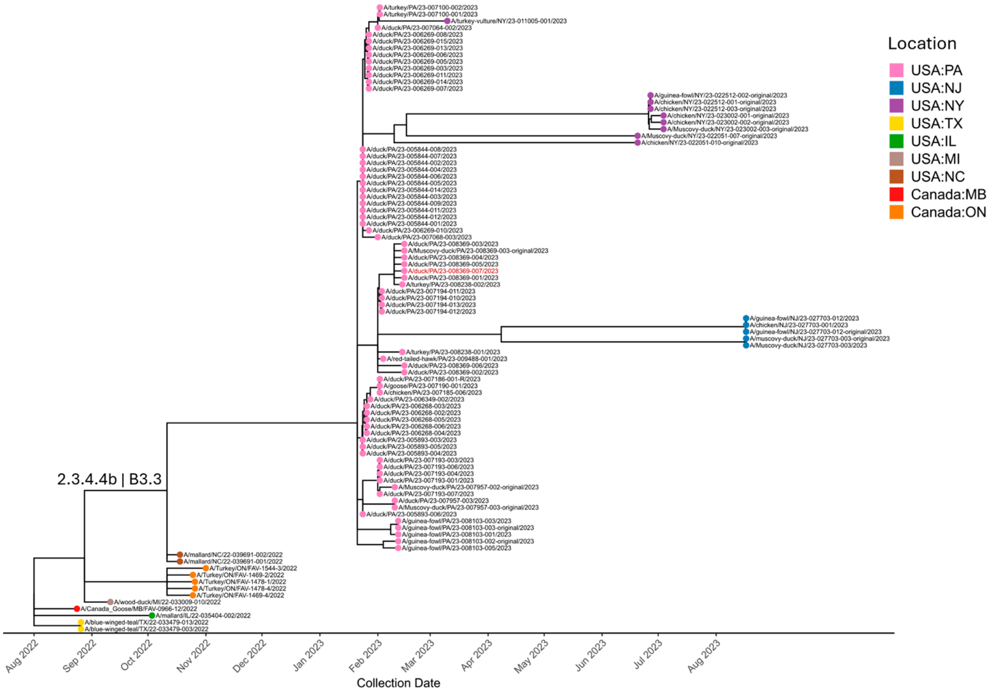

Supplementary Figure S4. **Nucleoprotein (NP) phylogenetic tree.** Time-scaled maximum-likelihood phylogenetic tree of the NP gene segment. The red color label indicates the reference isolate (23-008369-007).

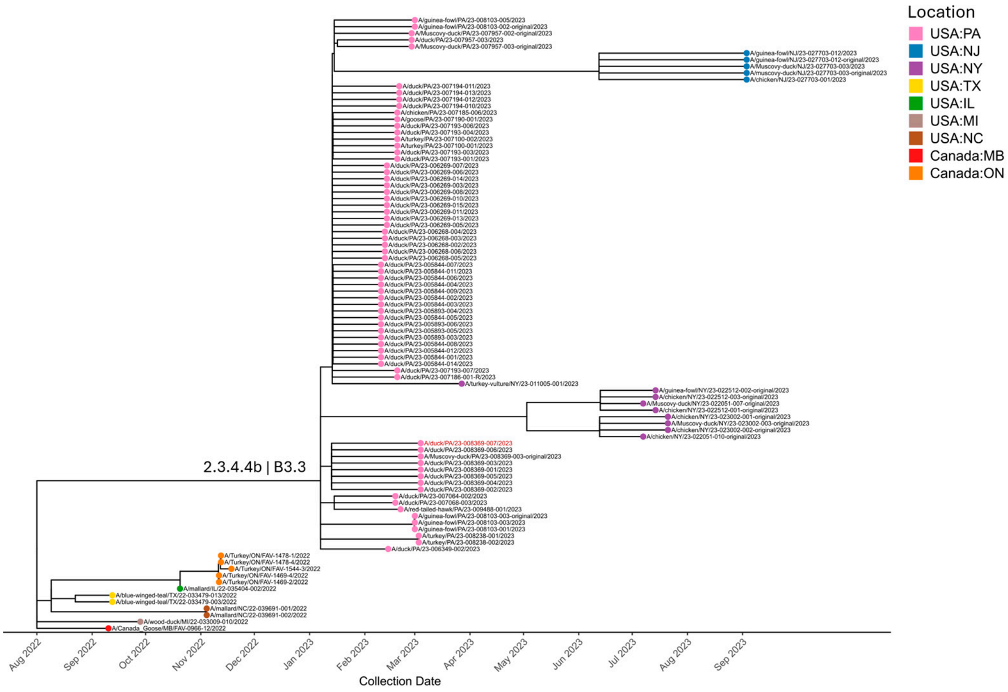

Supplementary Figure S5. NS (Non-Structural) **phylogenetic tree.** Time-scaled maximum-likelihood phylogenetic tree of the NS gene segment. The reference isolate (23-008369-007) is shown in red.

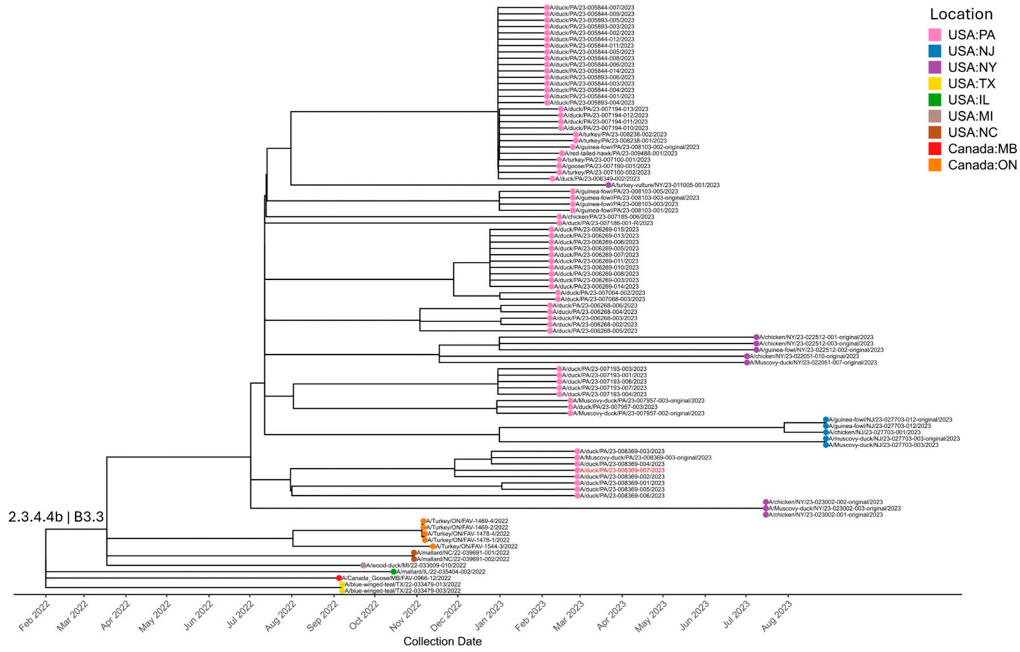

Supplementary Figure S6. **PA (Polymerase Acidic) Phylogenetic tree.** Time-scaled maximum-likelihood phylogenetic tree of the PA gene segment. The reference isolate (23-008369-007) is shown in red color.

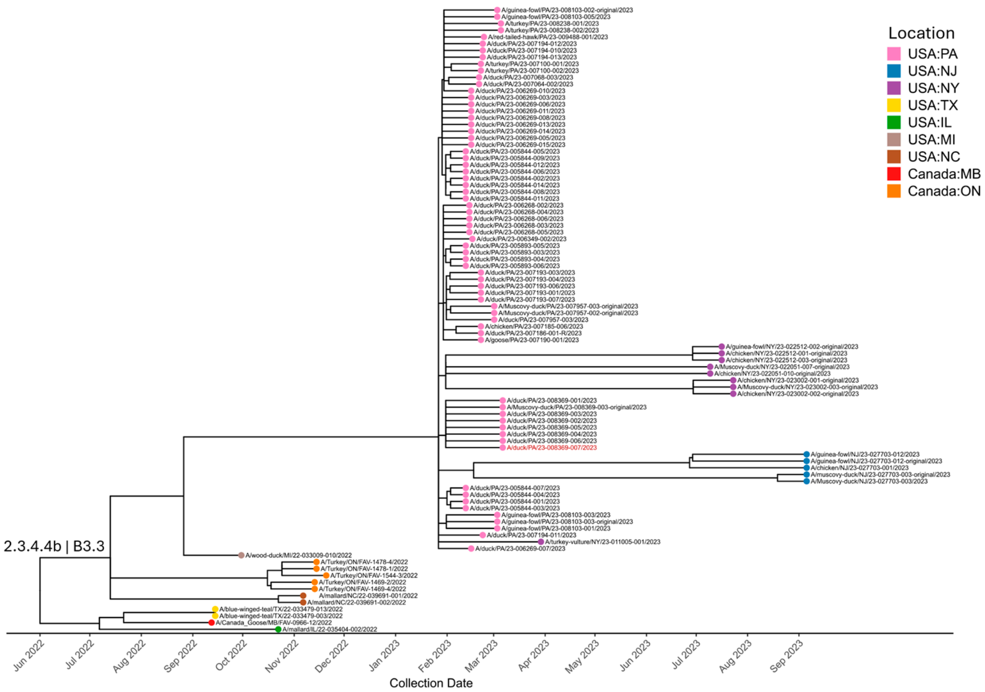

Supplementary Figure S7. PB1 (Polymerase Basic 1) Phylogenetic tree. Time-scaled maximum-likelihood phylogenetic tree of the PB1 gene segment. The reference isolate (23-008369-007) in red color shows in red color.

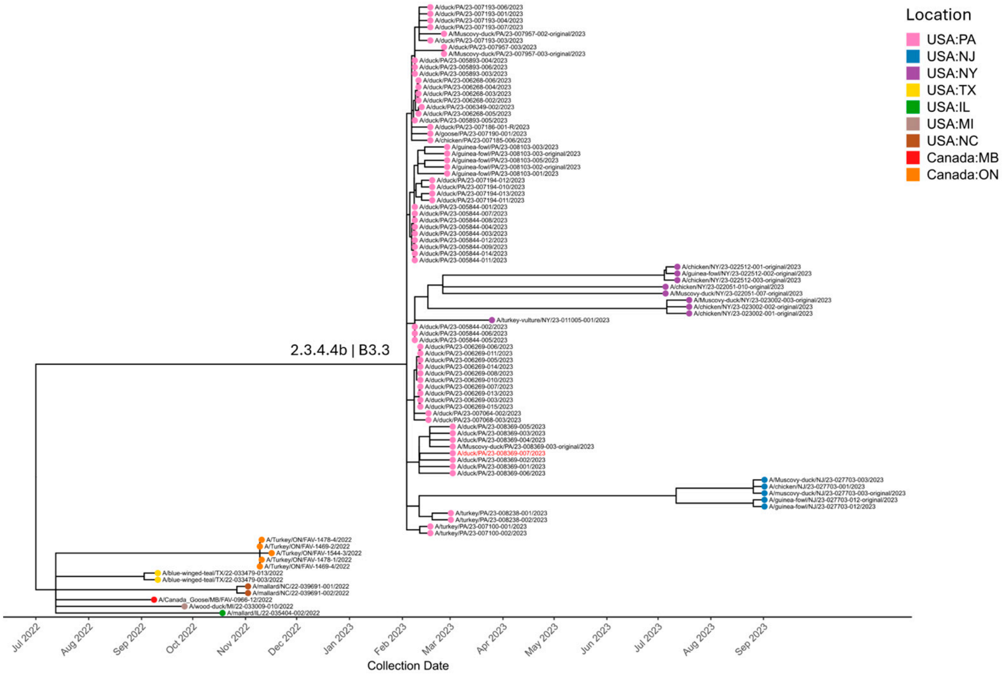

Supplementary Figure S8. PB2 (Polymerase Basic 2) Phylogenetic tree. Time-scaled maximum-likelihood phylogenetic tree of the PB2 gene segment. The red node label indicates the reference isolate (23-008369-007).

Supplementary Table S2: Vsnp analysis of avian influenza isolates genotype B3.3: The vSNP analysis and accompanying SNP tables display closely related isolates and identification of mixed SNPs. The columns identify the genome location of the SNP calls and the isolates are contained within the rows. The reference is listed across the top, identified as the "reference call". SNPs that are not highlighted match the reference. The map-quality scores measure confidence with maximum score of 60 for SNP correctly identification. The annotation of the SNP is provided at the bottom of the table.
